# Tumorendoprothetik

**DOI:** 10.1007/s00132-021-04151-3

**Published:** 2021-09-08

**Authors:** Reinhard Windhager

**Affiliations:** Universitätsklinik für Orthopädie und Unfallchirurgie, Medizinische Universität Wien, Währinger Gürtel 18–20, 1090 Wien, Österreich

Der Name Megaprothesen steht für Resektionsendoprothesen und wurde erstmals im Jahr 1976 anlässlich einer Publikation über den proximalen Femurersatz bei pathologischen Frakturen von van der Ghinst erwähnt [20]. Megaprothesen oder Resektionsendoprothesen haben eine lange Tradition bei der Rekonstruktion von Knochendefekten nach Tumoren und später auch bei posttraumatischen Defekten oder nach Revisionseingriffen.

Erste Versuche an unserer Klinik wurden 1964 mit dem Ersatz des distalen Femurs mittels einer Acrylharzprothese gestartet, die nach Resektion eines Riesenzelltumors eingesetzt wurde. Erwartungsgemäß war die Haltbarkeit dieser Prothesen limitiert. Der erste Einsatz im proximalen Femur erfolgte 1940 durch Austin Moore und Harald Pohlmann, die den Fall 1943 im amerikanischen *Journal of Bone and Joint Surgery* publizierten [12]. Bei all diesen Implantaten handelte es sich um Sonderanfertigungen, so wie die 1968 an unserer Klinik eingesetzte Resektionsendoprothese am proximalen Femur nach einem periostalen Osteosarkom, die für 48 Jahre ohne Revision funktionsfähig blieb.

**Figure 1:**
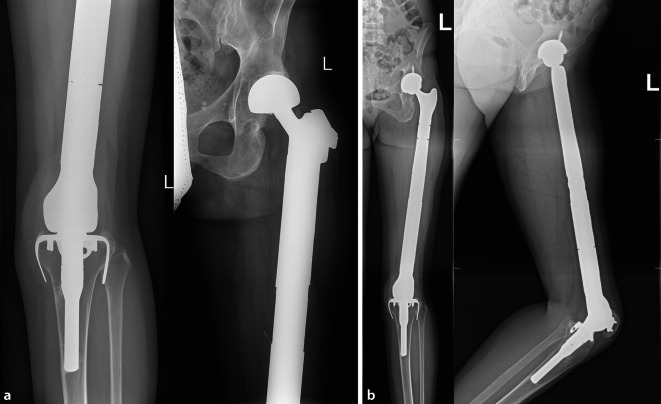

Anfang der 1970er-Jahre wurde neben dem proximalen Femur und Humerus auch der distale Femurersatz forciert, wobei erste Publikationen mit größeren Kasuistiken aus England stammen [1]. Beflügelt durch die Ergebnisse mit den individuell angefertigten Prothesen, wurde 1982 erstmals ein modulares Resektionssystem durch Rainer Kotz eingeführt und 1986 publiziert [9]. Dieses Kotz-Modulare-Femur-Tibia-Rekonstruktionssystem ermöglichte eine unmittelbare Verfügbarkeit im Operationsaal und damit auch eine deutliche Ausweitung der Indikationsbreite (Abb. 1a,b). So wurden die Resektionssysteme in weiterer Folge nicht nur bei Tumorprothesen, sondern auch – wie bereits erwähnt – bei ausgedehnten posttraumatischen Knochendefekten oder Knochensubstanzverlusten nach multiplen Revisionsoperationen eingesetzt.

Eine gleiche Entwicklung, wenn auch sogar schon früher, ist für die Resektionsprothesen an der oberen Extremität zu verzeichnen. Hervorzuheben ist, dass durch Martin Salzer bereits 1972 ein modulares Resektionssystem aus Keramik für die obere Extremität entwickelt worden ist und bis 1981 in Verwendung war [14]. Weitere Entwicklungen basierten auf Metallkomponenten und waren hinsichtlich intramedullärer Verankerung sowie Weichteilanbindung den Systemen in der unteren Extremität sehr ähnlich. Die Weichteilinsertion an den Prothesen ist jedoch ein dauerhaftes Problem, das bis dato noch nicht zufriedenstellend gelöst wurde. Durch Einführung von textilen Implantaten [3], die schlauchförmig über die Prothesen gezogen wurden, ist zwar die Insertion der Weichteile gewährleistet, allerdings die Gefahr der Infektion durch weiteres Fremdmaterial gesteigert. Während dies in der Primärsituation statistisch nicht zum Tragen kommt, sind bei Revisionsoperationen und Einsatz von textilen Implantaten erhöhte Infektionsraten zu beobachten [8].

Ein ungelöstes Problem stellt die deutlich erhöhte Infektionsrate von Resektionsendoprothesen dar, die um 10 % liegt und je nach Beobachtungsdauer über 15 % ansteigen kann [18]. Die Einführung von silberbeschichteten Prothesen hat anfänglich zu einer Euphorie geführt [2], die jedoch bei längerer Beobachtungsdauer einer Ernüchterung gewichen ist [16, 17]. Auch bei den Revisionen von infizierten Megaprothesen ist es im Laufe der Zeit zu einer Abnahme der Erfolgsrate gekommen, sodass in Zusammenschau mit den über einen längeren Zeitraum ansteigenden Infektionsraten von einer allgemeinen Resistenzentwicklung gegen Antibiotika auszugehen ist.

Einen Sonderstatus nehmen Wachstumsprothesen ein, die bereits 1976 in Stanmore implantiert wurden. Die verschiedenen Mechanismen der Verlängerung durch Insertion [15] von Ringen oder aber auch Austausch von modularen Segmenten, bis hin zum Einbringen von kugelförmigen Platzhaltern in ein teleskopartig expandierbares System, wurden durch Systeme, die über eine Spindel expandiert werden können, abgelöst. Die Verlängerung erfolgte anfangs minimal-invasiv durch Steckschraubenschlüssel, mit deren Hilfe die Prothesen um durchschnittlich 8–10 mm verlängert wurden. Da diese Mehrfacheingriffe nicht nur eine Belastung für die Patienten darstellten, sondern auch die Infektionsrate erhöhten, wurden nicht invasiv verlängerbare Prothesen entwickelt, von denen die mittels Magnetspule expandierbaren Prothesen von Stanmore derzeit am häufigsten in Verwendung sind. Nachteil dieser Prothesen ist die eingeschränkte Gelenkbeweglichkeit, bedingt durch die relative Verkürzung der Muskulatur nach der Verlängerung. Das von unserer Klinik entwickelte, nichtinvasive Verlängerungssystem berücksichtigt diese Muskelspannung, indem diese wie ein Regulator für die Verlängerung, die durch die Flexion der Prothese bewerkstelligt wird, funktioniert [10, 22].

Ein dauerhaftes Problemfeld in der endoprothetischen Versorgung sind Beckendefekte vor allem, wenn sie mit extensiven Weichteilresektionen nach malignen Tumoren einhergehen [13]. Die Anwendung von computerunterstützen, individuellen Implantaten hat bereits eine Tradition von mehr als 30 Jahren. Die Herstellung der Prothesen wurde anhand eines dreidimensionalen Fräsmodelles aus Computertomographiedaten bewerkstelligt, nachdem die Resektion und damit der Knochendefekt vom Chirurgen definiert worden ist. Mit herkömmlichen Gussverfahren wurden individuelle Metallimplantate produziert, die schließlich ohne weitere Adaptierungsfähigkeit in den Defekt eingebracht wurden und somit auf die Genauigkeit der Osteotomiedurchführung angewiesen waren. Eine Verbesserung der Passform durch Übereinstimmung des intraoperativ gesetzten Defektes und der geplanten Prothese erfolgte schließlich durch die Einführung von patientenspezifischen Resektionslehren und später durch die Verwendung der Navigation [23].

Als herausstechender Nebeneffekt dieser aufwendigen Prozedur konnte die Tatsache herausgearbeitet werden, dass durch die genaue Planung und die Erstellung eines dreidimensionalen Modelles die intraoperative Orientierung wesentlich besser war und damit die Anzahl der adäquaten Resektionsgrenzen signifikant erhöht werden konnte [21]. Dies wurde erst recht durch die patientenspezifischen Resektionslehren und die später eingeführte Navigation, inklusive Fusionsbilder gewährleistet. Obwohl die funktionellen Ergebnisse und die Stabilität von Anbeginn ausgezeichnet waren, war doch im längeren Beobachtungszeitraum die Infektionsrate überdurchschnittlich hoch, sodass mehr und mehr von der endoprothetischen Rekonstruktionsform im Becken Abstand genommen wurde. Auch die Einführung der Sockelprothesen brachte nicht den gewünschten Erfolg [6], in Besonderem war bei Revisionseingriffen und Beckendiskontinuität die Lockerungsrate außergewöhnlich hoch [19]. Die Indikation für Beckenprothesen wurde somit auf Patienten ohne neoadjuvante Chemotherapie oder Strahlentherapie reduziert, sodass bei primär malignen Knochentumoren in erster Linie Patienten mit Chondrosarkomen herfür infrage kommen.

Erfolgreicher waren individuelle Beckenimplantate bei Revisionsoperationen, wo sie durch die Einführung des 3‑D-Laser-Metall-Druckverfahrens und des damit verbundenen verkürzten Herstellungsverfahrens eine weite Verbreitung erfuhren. Diese Drucktechnologie ermöglichte auch, die Oberfläche osteointegrativer zu gestalten, als dies mit herkömmlichen Gussverfahren inklusive Nachbearbeitungstechniken möglich war [23]. Auch die Beschichtung mit Silber hat zu keiner signifikanten Änderung der Infektionsrate geführt, sodass diese spezielle Form der megaprothetischen Versorgung am Becken in erster Linie Revisionsoperationen vorbehalten bleibt.

Eine Thematik, die bei Megaprothesen und jungen Patienten immer wieder auftaucht, ist die Sportfähigkeit. Während bei konventionellen Endoprothesen der Hüfte und des Kniegelenkes über die Jahrzehnte hinweg ein deutlicher Wandel hin zu mehr sportlicher Betätigung eingetreten ist, herrscht im Bereich der Resektionsendoprothetik noch deutliche Zurückhaltung. Eigene Analysen haben gezeigt, dass Patienten unabhängig von Empfehlungen oder Verboten Sport ausüben und die Rate der Wiederaufnahme der sportlichen Betätigung ähnlich wie bei den konventionellen Endoprothesen liegt, wobei die unterschiedliche Altersstruktur berücksichtigt werden muss. Eigene Analysen haben gezeigt, dass die funktionelle Aktivität, sowohl beim proximalen Femur als auch bei Megaprothesen des Kniegelenkes eine Zunahme der sportlichen Aktivität bis 5 Jahre postoperativ verzeichnen lässt [7, 11].

Dieses ist deutlich im Gegensatz zur konventionellen Endoprothetik, wo das Maximum der Funktion nach einem Jahr erreicht ist und danach nur eine geringe Zunahme zu verzeichnen ist.

Ein wohl wesentlicher Beitrag für die Vergleichbarkeit der verschiedenen Megaprothesensysteme, die am Markt sind, war die Einführung einer gemeinsamen Sprache in Bezug auf Komplikationen und Reoperationen. Das von uns wesentlich mitgestaltete Klassifikationssystem unterscheidet mechanische Komplikationen, wie Weichteileversagen, aseptische Lockerung und strukturelles Versagen sowie nichtmechanische Komplikationen, wie Infektionen oder lokale Tumorprogression [4]. Für den Extremitätenerhalt wurde dieses Rekonstruktionssystem in den einzelnen Kategorien noch weiter differenziert, sodass auch für Allograft-Rekonstruktionen und pädiatrische Versorgungen, wie Wachstumsprothesen, eine gleiche Sprache gegeben ist und somit in Zukunft die Vergleichbarkeit der Ergebnisse nachvollziehbar gewährleistet ist [5].

Trotz der mannigfaltigen Erfolge sind dennoch die Aufgaben für die Zukunft klar umrissen, als es gilt, einerseits die Infektionsraten zu senken, sei es durch Änderungen der Oberflächenbeschichtung oder aber im chirurgischen Management, und andererseits die Insertion der Weichteile im Besonderen der Sehnen an den Prothesen zu verbessern.
